# Synthesis and Study of Optical Characteristics of Ti_0.91_O_2_/CdS Hybrid Sphere Structures

**DOI:** 10.1186/s11671-018-2488-3

**Published:** 2018-03-07

**Authors:** Lingbin Kong, Qinfeng Xu, Meng Zhang, Dehua Wang, Mingliang Liu, Lei Zhang, Mengmeng Jiao, Honggang Wang, Chuanlu Yang

**Affiliations:** School of Physics and Optoelectronic Engineering, Ludong University, Yantai, 264025 China

**Keywords:** Hybrid spherical structures, Ti_0.91_O_2_ nanosheets, CdS nanoparticles, Indirect optical transitions

## Abstract

The optical properties of alternating ultrathin Ti_0.91_O_2_ nanosheets and CdS nanoparticle hybrid spherical structures designed by the layer-by-layer (LBL) assembly technique are investigated. From the photoluminescence (PL) spectral measurements on the hybrid spherical structures, a spectrum-shifted fluorescence emission occurs in this novel hybrid material. The time-resolved PL measurements exhibit a remarkably increased PL lifetime of 3.75 ns compared with only Ti_0.91_O_2_ spheres or CdS nanoparticles. The novel results were attributed to the enhanced electron-hole separation due to the new type II indirect optical transition mechanism between Ti_0.91_O_2_ and CdS in a charge-separated configuration.

**Electronic supplementary material:**

The online version of this article (10.1186/s11671-018-2488-3) contains supplementary material, which is available to authorized users.

## Background

Semiconductor composite nanostructures have attracted more attention due to the optimal assembling of conduction band and valance band for the photovoltaic applications and other optoelectronic devices [1–4]. Spatial separation of the electron and hole in the type II semiconductor composite nanostructures can result in a prolonged lifetime of charge carriers which has desirable optical characteristics for applications such as light sources [5, 6], lasers [7–9], and photovoltaic devices [10, 11]. Many studies of indirect optical transition (IOT) effect in the type II composite nanostructures have been reported over the past few years. For instance, IOT phenomenon has been reported in ultrathin hybrid sphere nanostructures including graphene oxide and TiO_2_ nanosheets [12] or coupled quantum dots system [13]. In recent years, TiO_2_ is an important optical materials which has been widely investigated owing to its outstanding optical properties for use in photocatalysis and solar cells, but the wide bandgap (3.2 eV) of TiO_2_ limits its photocatalytic property in the UV region. In order to extensively exploit the optical activity in the visible light region, the surface of TiO_2_ nanosheets coated with quantum dots has been investigated as a superior alternative for dye-sensitized solar cell [14–18]. Particularly important, the composite system of TiO_2_ nanosheets coupled with CdS quantum dots (QDs) has been widely studied for various applications due to its suitable bandgap (2.4 eV) and excellent optical properties [19–21]. Combining these features, the TiO_2_/CdS hybrid structures have been recently highlighted as a unique system [22–26]. Moreover, the CdS nanoparticles coated with TiO_2_ nanosheets can greatly improve its optical activity. So far, exciton separation and carrier extraction are the major bottleneck achieving highly efficient material-sensitized solar cells. However, fundamental studies on photoexcited carrier dynamics based on TiO_2_/CdS hybrid spheres are limited. Therefore, the photoluminescence (PL) properties and time-resolved PL decays of composite nanostructures consisting of alternating Ti_0.91_O_2_ nanosheets and CdS nanoparticles are investigated in this paper. From the PL spectra and time-resolved PL decay measurements, the new type II indirect optical transition contributes to clarify the novel fluorescence emission mechanism of composite nanostructures consisting of Ti_0.91_O_2_ nanosheets and CdS nanoparticles that are different from traditional TiO_2_/CdS fluorescence radiative transition systems. The excitation power- and excitation wavelength-dependent PL spectra and time-resolved PL decay measurements were also further investigated to affirm the recombination properties of charge carriers and elucidate the competition mechanism of different radiative transition pathways in Ti_0.91_O_2_/CdS composite nanostructure. These novel results provide a useful viewpoint for the design of charge separation and charge extraction in TiO_2_ and CdS composite nanostructures for various optoelectronic device applications.

## Methods

### Synthesize Samples

The synthesis of Ti_0.91_O_2_ nanosheets and CdS nanoparticles has been reported based on the layer-by-layer self-assembly technique [27]. The overall procedure for fabricating multilayer Ti_0.91_O_2_/CdS composite nanostructures is demonstrated as follows: the poly(methly methacrylate) (PMMA) solid spheres were completely diluted by the protonic polyethylenimine (PEI) aqueous solution, in order to ensure the saturated adsorption of PEI on PMMA solid spheres surfaces. The PMMA solid spheres coated with PEI are diluted with deionized water by ultrasonic treatment; then, negatively charged Ti_0.91_O_2_ nanosheets were added to the hybrid PMMA coated with PEI solution under stirring, the PMMA combine with Ti_0.91_O_2_ nanosheets due to the interior electrostatic interaction of the opposite charge. The above procedure was repeated. The multilayer PEI/Ti_0.91_O_2_/PEI/CdS hybrid sphere nanostructures that have been deposited onto PMMA spheres were achieved based on the above repeated synthesis procedures. During microwave irradiation, PEI moiety was removed and PMMA particles were decomposed. After reaction, hollow spheres consisting of alternating Ti_0.91_O_2_ nanosheets and CdS QDs were obtained, and trifle PMMA residue was removed with tetrahydrofuran (THF). Finally, the hybrid hollow spheres with multilayer Ti_0.91_O_2_/CdS nanostructures were obtained.

### Experiment Apparatus

The sample images of solid Ti_0.91_O_2_/CdS hybrid spheres and hollow Ti_0.91_O_2_/CdS hybrid spheres were measured by transmission electron microscopy (TEM) and scanning electron microscopy (SEM), respectively. The appropriate amounts of solid Ti_0.91_O_2_/CdS hybrid spheres and hollow Ti_0.91_O_2_/CdS hybrid spheres were diluted by deionized water to have lower sample densities. Diluted samples were spin-coated on silica coverslip to prepare thin films for optical measurement with the 266 and 400 nm excitation. The optical measurements of all samples were carried out at room temperature. For PL spectral measurements, the 800 nm ps Ti:Sapphire laser with 76 MHz repetition rate was used to generate the 266 and 400 nm wavelength pulse laser based on second-harmonic and third-harmonic conversion technique, respectively. Two hundred sixty-six nanometer and 400 nm pulse laser at an incident angle of ~ 45° relative to the vertical direction was focused onto the sample surface with a power density of ~ 100 W/cm^2^. The PL from samples was collected vertically by a ×60 objective and sent to the spectrometer, and the emission PL spectra were recorded with a monochromator (Acton SP-2500i, 0.5 m, 150 lines mm^− 1^ grating, blazed at 500 nm) fitted with a Princeton Instruments liquid-nitrogen-cooled charge-coupled device (CCD) camera. For time-resolved PL decay measurements, the PL from the samples was collected by the same objective and then detected by the single photon counting system with the 250 ps time resolution. Moreover, the corresponding 450, 500, and 550 nm band-pass filter with a 10-nm bandwidth was used to effectively measure the different wavelength PL lifetimes.

## Results and Discussion

**Fig. 1:**
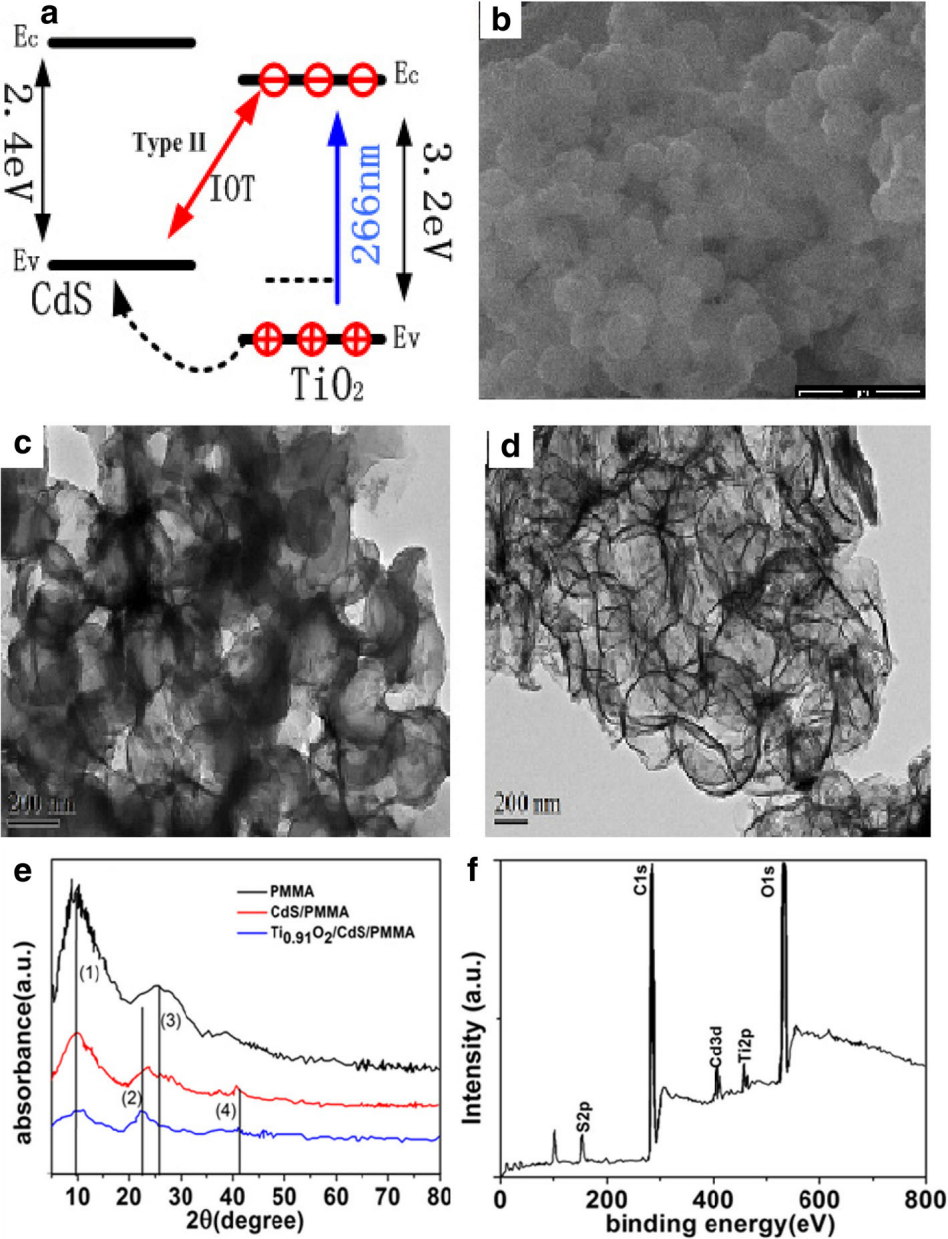
**a** Energy band diagram of hybrid spheres Ti_0.91_O_2_ and CdS. **b** Scanning electron microscopy (SEM) images of Ti_0.91_O_2_/CdS. **c** Transmission electron microscopy (TEM) images of the solid Ti_0.91_O_2_/CdS hybrid spheres. **d** Hollow Ti_0.91_O_2_/CdS hybrid spheres. **e** XRD of PMMA, CdS, and Ti_0.91_O_2_/CdS. **f** XPS spectrum of Ti_0.91_O_2_/CdS

Figure 1a shows energy levels of Ti_0.91_O_2_ nanosheets and CdS nanoparticles, and the CdS nanoparticles have a higher conduction band level compared with that of Ti_0.91_O_2_ nanosheets. The scanning electron microscopy (SEM) image of the hybrid spheres Ti_0.91_O_2_ nanosheets and CdS nanoparticles with several hundred nanometers length and smooth surfaces are shown in Fig. 1b. The transmission electron microscopy (TEM) images of the solid Ti_0.91_O_2_/CdS hybrid spheres and hollow Ti_0.91_O_2_/CdS composite sphere nanostructures are shown in Fig. 1c, d, respectively. Figure 1a shows the XRD patterns of pure PMMA, CdS, and Ti_0.91_O_2_/CdS film. Compared to pure PMMA, Ti_0.91_O_2_/CdS and CdS film exhibits new peaks 2 and 4 indicating the presence of the cubic phase CdS. The composition of Ti_0.91_O_2_/CdS was identified by e X-ray photoelectron spectroscopy (XPS), as shown in Fig. 1f. Therefore, hollow spheres consisting of alternating Ti_0.91_O_2_ nanosheets and CdS QDs were obtained. In order to further verify synthesis of Ti_0.91_O_2_ nanosheets and CdS nanoparticles based on the layer-by-layer self-assembly technique, the absorption spectra and Raman spectra of Ti_0.91_O_2_ and Ti_0.91_O_2_/CdS are shown in Additional file 1: Figure S1 and Figure S2, respectively. Compared with Raman spectra of Ti_0.91_O_2_ nanosheets, the Raman spectra of Ti_0.91_O_2_/CdS demonstrate a combination of Ti_0.91_O_2_ nanosheets and CdS nanoparticles.

**Fig. 2:**
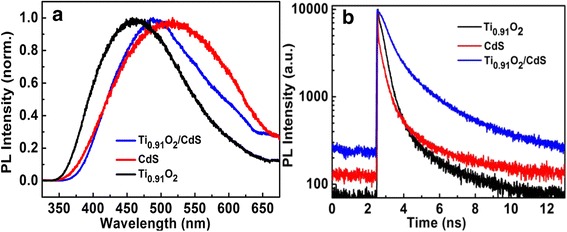
**a** PL spectra of the Ti_0.91_O_2_(black), CdS(red), and Ti_0.91_O_2_/CdS (blue) samples excited at 266 nm. **b** PL decay curves of the Ti_0.91_O_2_(black), CdS(red), and Ti_0.91_O_2_/CdS (blue) samples with the 266 nm excitation

The photoluminescence (PL) spectra of Ti_0.91_O_2_ (black), CdS (red), and Ti_0.91_O_2_/CdS (black) excited at 266 nm are shown in Fig. 2a. The fluorescence peaks of Ti_0.91_O_2_ and CdS are around 450 and 530 nm with the 266 nm excitation, respectively. Because the bandgap energy of TiO_2_ is 3.2 eV, the red-shifted PL spectra observed in Fig. 2a should attribute to defect levels generated inside the bandgap of Ti_0.91_O_2_ so that holes generated in the Ti_0.91_O_2_ valence band can relax to different defect state levels by the nonradiative channels and then recombine with the electrons of Ti_0.91_O_2_, giving rise to the related defect state optical emission. Under the 266 nm excitation, the fluorescence emission peak around 530 nm from CdS nanoparticles embodies smaller energy bandgap than that of CdS (2.48 eV). We suppose that the nonradiative transition of excited electrons from the conduction band bottom to different defect states level occurs in the CdS nanoparticles. However, the fluorescence emission peak shifts to 500 nm when the Ti_0.91_O_2_/CdS hybrid structure excited at 266 nm. If we exclude the contribution of either Ti_0.91_O_2_ or CdS to the blue-shifted spectra emission; then, this fluorescence mechanism attributes to an indirect optical transition (IOT) in hybrid interface of Ti_0.91_O_2_/CdS system. In the traditional type II TiO_2_/CdS composite nanostructure, light excitation of TiO_2_ and CdS will transfer electrons from the higher conduction band of CdS to the lower conduction band of TiO_2_ and generated holes from the lower value band of TiO_2_ to the higher value band of CdS nanoparticles. If the whole PL emission of Ti_0.91_O_2_/CdS hollow spheres comes from CdS nanoparticles, we should observe the faster PL decay process caused by a nonradiative decay channel that the electrons transfer from the conduction band of CdS nanoparticles to the conduction band of TiO_2_ due to the fluorescence quenching effect as in traditional TiO_2_/CdS system. Therefore, a new electron transfer mechanisms were proposed for the present Ti_0.91_O_2_/CdS hybrid nanostructure hollow sphere system: the electrons in the conduction band of Ti_0.91_O_2_ recombine with holes in the valence band of CdS nanoparticles; then, the spectra-shifted emission emerges in this Ti_0.91_O_2_/CdS composite material.

To better study the charge transfer between present Ti_0.91_O_2_ nanosheets and CdS nanoparticles, transient time-resolved PL decay measurements were carried out on the samples excited at 266 nm. The PL decay curves can be well fitted to the biexponential function in the form of *f*(*t*) = *A*_1_ exp(−*t*/*τ*_1_) + *A*_2_ exp(−*t*/*τ*_2_). The average lifetime τ is calculated by the form of *τ* = (*A*_1_*τ*_1_^2^ + *A*_2_*τ*_2_^2^)/(*A*_1_*τ*_1_ + *A*_2_*τ*_2_) and the all later lifetime calculations base on the form. Therefore, the average PL lifetime for Ti_0.91_O_2_ is 0.43 ns and the average PL lifetime for CdS is 0.35 ns as shown in Fig. 2b. More importantly, the average PL lifetime of Ti_0.91_O_2_/CdS hybrid structures is remarkably increased to 3.75 ns compared with the abovementioned PL lifetime of only Ti_0.91_O_2_ nanosheets or CdS nanoparticles. Based on the new type charge transfer mechanisms in Ti_0.91_O_2_/CdS hybrid interfaces, the electrons stay at the conduction band of Ti_0.91_O_2_ nanosheets, but the holes can either relax to the defect state levels or be transferred to the valence band levels of CdS nanoparticles. Due to the lower symmetry at the Ti_0.91_O_2_/CdS hybrid interface, the optical recombination from the electrons in the conduction band of Ti_0.91_O_2_ and the holes in the value band level of CdS causes prolonged PL lifetime. However, the experiment results also indicate weak optical activity of Ti_0.91_O_2_/CdS hollow sphere nanostructures under a 400-nm laser excitation and no obvious occurring of sensitization of CdS on Ti_0.91_O_2_. This means that the electrons in the conduction band of CdS would be inclined to recombination with hole in the value band of CdS rather than transfer to the conduction band of Ti_0.91_O_2_ nanosheets. These experiment results show that different from traditional type II fluorescence can be well explained by the new type II spatial separation of electrons and holes across the Ti_0.91_O_2_/CdS hybrid interface. Moreover, to better compare charge transfer and electronic interaction between Ti_0.91_O_2_/CdS and TiO_2_/CdS, PL spectra and transient time-resolved PL decay measurements were carried out on the samples Ti_0.91_O_2_/CdS and TiO_2_/CdS excited at 266 nm laser wavelength as shown in Additional file 1: Figure S3(a). Compared with TiO_2_/CdS spheres, the emission peak of Ti_0.91_O_2_/CdS spheres show the same emission peak. However, the prolonged decay lifetime observed in Ti_0.91_O_2_/CdS hollow spheres reveals that decay dynamics for Ti_0.91_O_2_/CdS hollow spheres are fundamentally different from traditional TiO_2_/CdS system.

**Fig. 3:**
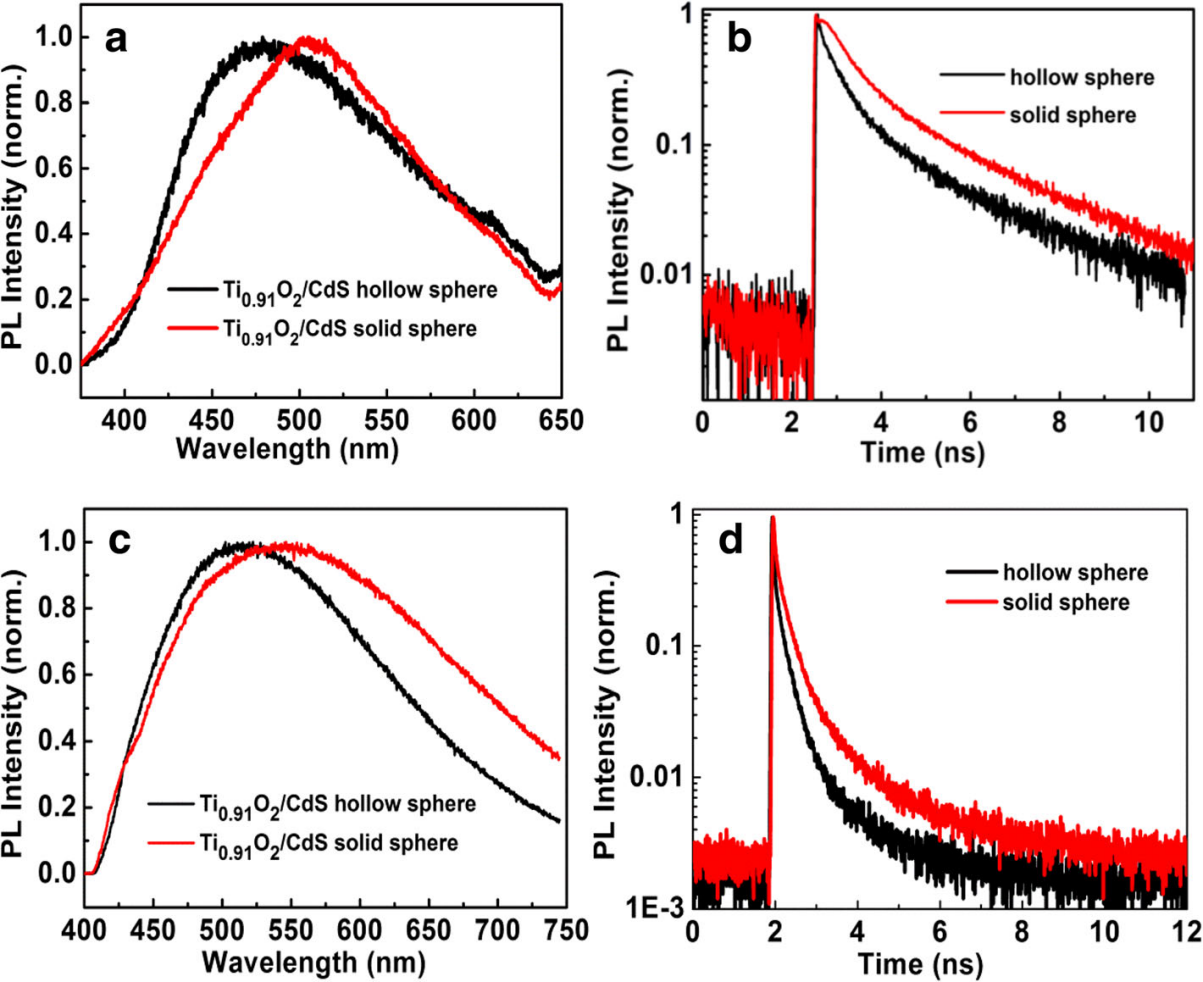
**a** PL spectra of hollow Ti_0.91_O_2_/CdS (black) and solid Ti_0.91_O_2_/CdS (red) samples excited at 266 nm. **b** PL decay curves of hollow Ti_0.91_O_2_/CdS (black) and solid Ti_0.91_O_2_/CdS (red) samples with the 266 nm excitation. **c** PL spectra of hollow Ti_0.91_O_2_/CdS (black) and solid Ti_0.91_O_2_/CdS (red) samples excited at 400 nm. **d** PL decay curves of hollow Ti_0.91_O_2_/CdS (black) and solid Ti_0.91_O_2_/CdS (red) samples with the 400 nm excitation

To further investigate the interactive charge transfer mechanism between CdS and Ti_0.91_O_2_ hybrid structure, we compare the PL spectra and PL decay properties of hollow and solid Ti_0.91_O_2_/CdS hybrid spheres with 266 and 400 nm excitation, respectively. When Ti_0.91_O_2_/CdS is excited at 266 nm, the electrons ultimately stay at the conduction band of Ti_0.91_O_2_, and the holes can be transferred to the value band of CdS nanoparticles. The optical recombination between electrons in the conduction band of Ti_0.91_O_2_, and holes in the value band of CdS is allowed. However, the Ti_0.91_O_2_/CdS solid spheres contain the PMMA template and the PEI moiety; thus, these insulating organic surfactants hinder charge transport in the Ti_0.91_O_2_/CdS interface. Due to the electronic coupling between the CdS and Ti_0.91_O_2_ hybrid structure, the charge carrier mobility can be greatly enhanced by removing the organic surfactants from the quantum dots (QDs) surface. The photoluminescence (PL) spectra and PL decay lifetime are shown in Fig. 3a, b, respectively. The PL peaks of Ti_0.91_O_2_/CdS solid spheres were red-shifted compared with Ti_0.91_O_2_/CdS hollow sphere, and the average PL lifetime is 4.25 ns (solid spheres) and 3.69 ns (hollow sphere), which implies the photoexcited holes in the valence band of Ti_0.91_O_2_ is more difficult to inject into the valence band of CdS in solid hybrid structures. The PMMA templates and PEI were completely eliminated to enhance inter-connectivity between alternating nanosheets of CdS and Ti_0.91_O_2_ and lead to an enhanced PL quenching phenomenon and shortened PL decay lifetime. Thus, the PL quenching effect in Ti_0.91_O_2_/CdS hybrid structures is attributed to the electrons dissociation because the bleach decay of the surface trapping does not explain the efficient PL quenching phenomenon. The charge separation process in Ti_0.91_O_2_/CdS hybrid structures occurs via the hole transfer from the valence band of Ti_0.91_O_2_ to the valence band of CdS nanocrystals based on the new type II indirect optical transition in a close-packed Ti_0.91_O_2_/CdS hybrid nanostructures. Thus, the carrier recombination lifetime by indirect optical transition has been decreased from 4.25 ns (solid sphere) to 3.69 ns (hollow sphere).

By tuning the excitation wavelengths to 400 nm at higher excitation power, the PL spectra and transient time-resolved PL decay dynamics were measured. The results show that the feeble PL spectra with 10 times integrated time are shown in Fig. 3c, and the average PL lifetime (0.59 ns) of Ti_0.91_O_2_/CdS solid hybrid structures is shorter than the PL lifetime (0.45 ns) of Ti_0.91_O_2_/CdS hollow hybrid structures as shown in Fig. 3d, suggesting that CdS has higher electron transfer rate toward Ti_0.91_O_2_ according to the traditional type II Ti_0.91_O_2_/CdS heterostructure. Compared with the case of 266 nm excitation, the shorter PL lifetime with 400 nm excitation indicates that PL quenching effect is further enhanced due to optical recombination between electrons and holes in the Ti_0.91_O_2_/CdS system or the wastage of holes for photocorrosion in the CdS nanoparticles. Therefore, the Ti_0.91_O_2_/CdS hollow hybrid spheres show weak optical activity under a 400-nm laser excitation, and no obvious sensitization emerges in the Ti_0.91_O_2_/CdS hybrid spheres.

**Fig. 4:**
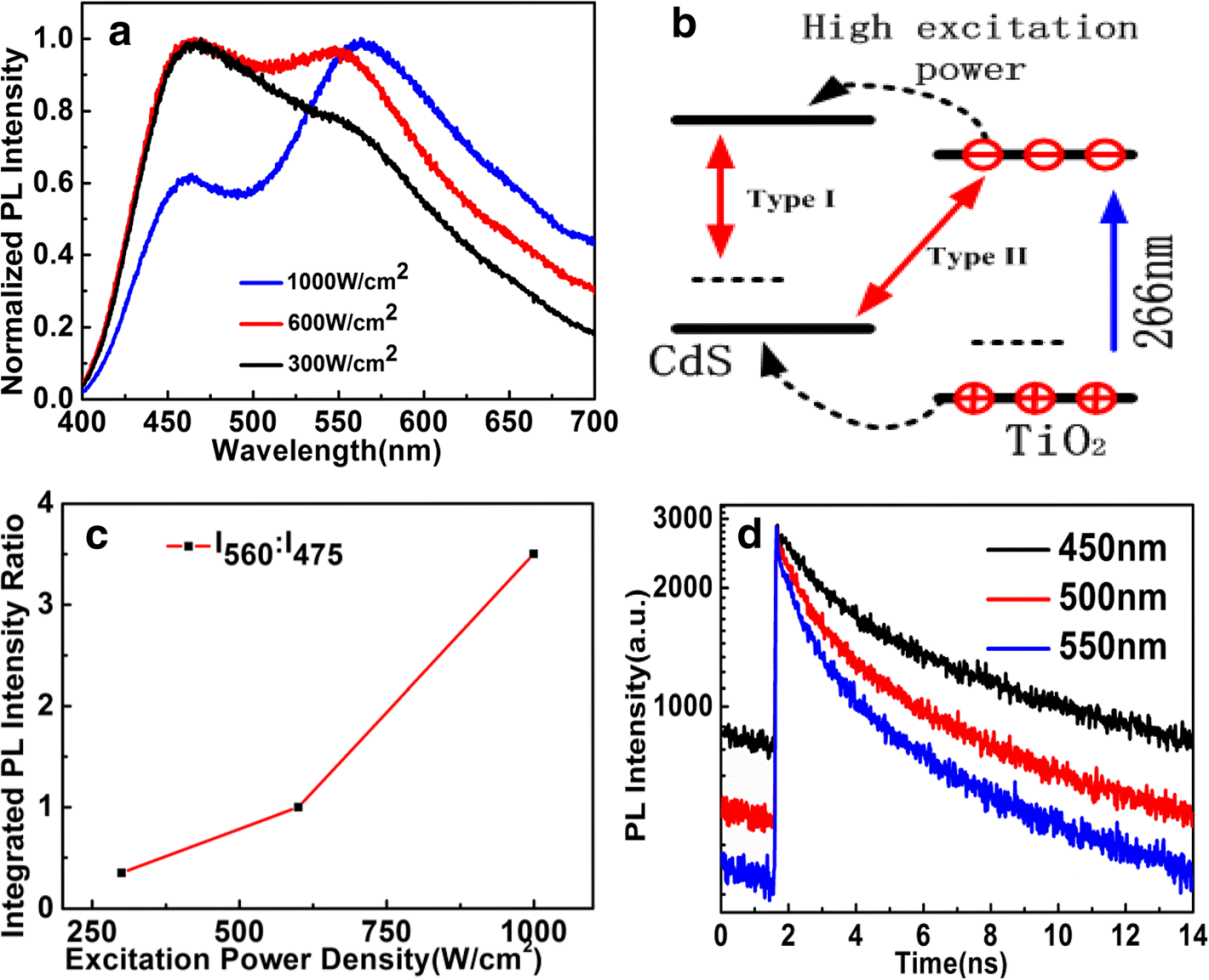
**a** Excitation power dependence of PL spectra. **b** Electron transfer from conduction band of Ti_0.91_O_2_ to CdS with high-power excitation. **c** The integrated PL intensity ratio between central wavelength 560 and 475 nm. **d** The time-resolved PL measurements for 450, 500, and 550 nm with 266 nm excitation wavelength

To further investigate the charge carrier relaxation pathways in Ti_0.91_O_2_/CdS hollow hybrid interface, the excitation intensity-dependent PL spectra in the Ti_0.91_O_2_/CdS hybrid spherical structures were investigated under a 266-nm laser excitation. Under a 266-nm low-excitation intensity, we first observed that the 475 nm peak is dominant in the PL spectrum. With increasing the excitation power, the corresponding PL spectra intensity varied as a function of the excitation power ranging from 300 to 1000 W/cm^2^ and the central peak wavelength of PL spectrum shift from 475 to 560 nm as shown in Fig. 4a. We tentatively attributed to electron transfer from conduction band of Ti_0.91_O_2_ to conduction band of CdS when Ti_0.91_O_2_/CdS hybrid nanostructures were excited by higher power 266 nm laser; then, the electron-hole recombination occurs between electrons in conduction band of CdS and holes in the valence band or the defect level of CdS nanoparticles according to type I recombination mechanism as shown in Fig. 4b. These varied PL spectra show that the red shift occurs with increasing excitation power. Such results confirm the different nature and origin of the emissions wavelength at 475 and 560 nm, respectively. Thus, the 475-nm emission wavelength indicates the type II emission property and the 560-nm emission wavelength reflects the type I emission property. The spectra shifted with excitation power indicate the competition mechanism between spatially direct and indirect recombination channels in Ti_0.91_O_2_/CdS composite interfaces. With the continuous by increasing the excitation power, more electrons with high-power excitation transfer from the conduction band of Ti_0.91_O_2_ to the conduction band of CdS nanoparticles, leading to a strongly increasing intensity ratio between central wavelength 560 and 475 nm, and the photoluminescence intensity ratio of two emission peaks can reach to 3.5 as shown in Fig. 4c. However, the weak photoluminescence intensity implies that the electron transfer from the conduction band of Ti_0.91_O_2_ to the conduction band of CdS nanoparticles only plays a minor role in the appearance of PL emission.

To further verify the two kinds of transition mechanisms with different excitation power in the Ti_0.91_O_2_/CdS hollow spheres, the probing wavelength-dependent time-resolved photoluminescence (TRPL) experiment was performed with different excitation power density. It is suitable to monitor the charge carrier transfer or electron-hole recombination process in the Ti_0.91_O_2_/CdS interface. The TRPL lifetimes of Ti_0.91_O_2_/CdS were measured with different probe wavelengths at 450, 500, and 550 nm, respectively. And the corresponding 450, 500, and 550 nm band-pass filter with a 10-nm bandwidth was used. The TRPL give longer decay lifetimes (3.72 ns) at shorter wavelength (450 nm) in the Ti_0.91_O_2_/CdS interface as shown in Fig. 4d because of the spatial separation of the charge carriers in the composite structures with the electrons in the conduction band of Ti_0.91_O_2_ nanosheets and holes in valence band of CdS nanoparticles. This type II hybrid structures reduce the PL intensity due to the smaller overlap between electron and hole wave functions and consequently enhances PL recombination lifetimes. However, the PL lifetimes (1.61 ns) at longer wavelength (550 nm) become faster due to enhancing the wave function overlap between the electron of conduction band (CB) and hole of valence band (VB) in the CdS nanoparticles as shown in Fig. 4d. This findings clearly reveal that the photoexcited carriers in the Ti_0.91_O_2_/CdS make a significant contribution to the longer PL lifetimes. This evidence further confirms that the dominant PL is from the recombination between the electron in the CB of Ti_0.91_O_2_ and hole in the VB in of CdS nanoparticles. These findings confirm that electrons in the conduction band of Ti_0.91_O_2_ nanosheets recombine with holes in the valence band of CdS nanoparticles through indirect optical transition that is different from traditional TiO_2_/CdS system. These prolonged carrier lifetime makes the Ti_0.91_O_2_/CdS composite nanostructure most suitable for photovoltaic applications. To characterize the ability of the synthetic samples, linear J−V curves were recorded as shown in Additional file 1: Figure S4. The great enhancement of the photocurrent after CdS sensitization shows the advantage of the Ti_0.91_O_2_/CdS compared to the Ti_0.91_O_2_ with light illumination. Therefore, a higher loading of the photosensitizer will lead to a higher photocurrent density.

## Conclusions

In summary, we have detected novel indirect optical transition (IOT) properties in the multilayer PEI/Ti_0.91_O_2_/PEI/CdS hybrid nanostructures from the PL spectra and time-resolved PL measurements. From the PL spectral and TRPL measurement, the red-to-blue shift light emission emerges in this novel composite material. And prolonged photoluminescence lifetime of Ti_0.91_O_2_/CdS composite nanostructure compared with only Ti_0.91_O_2_ spheres or CdS nanoparticles was found. These results demonstrate new photoluminescence recombination mechanism due to the optical recombination between holes in the value band level of CdS and electrons in the conduction band level of Ti_0.91_O_2_ that is different from traditional TiO_2_/CdS composite system. By tuning the excitation wavelengths and excitation power, the PL spectra and PL lifetimes of Ti_0.91_O_2_/CdS hybrid structures exhibit an excitation wavelength- and excitation power-dependent behavior. From the bandgap configurations, the IOT for Ti_0.91_O_2_/CdS hybrid structure which lead to prolonged carrier lifetime make for charge carrier separation and extraction for the important applications in photovoltaic system.

## Additional file

Additional file 1: Optical measurement of alternating ultrathin Ti_0.91_O_2_ nanosheets and CdS nanoparticles hybrid spherical structures by the layer-by-layer assembly technique. (DOC 593 kb)

## Abbreviations

IOT: Indirect optical transition
PL: Photoluminescence
PMMA: Polymethyl methacrylate
QDs: Quantum dots
TRPL: Time-resolved photoluminescence
