# Supplementary material for: Synthesis and Study of Optical Characteristics of Ti0.91O2/CdS Hybrid Sphere Structures
Source: Nanoscale Res Lett. 2018 Mar 7;13:80. doi: 10.1186/s11671-018-2488-3 (PMC5842163; doi:10.1186/s11671-018-2488-3)
Supplement: Supplementary file 1 — Optical measurement of alternating ultrathin Ti0.91O2 nanosheets and CdS nanoparticles hybrid spherical structures by the layer-by-layer assembly technique. (DOC 593 kb) [file 11671_2018_2488_MOESM1_ESM.doc]

Figure S1. displays the UV-vis absorption spectra of pure CdS, Ti_0.91_O_2_ and Ti_0.91_O_2_/CdS. Compared with the absorption spectra of pure CdS andTi_0.91_O_2_, there is an obvious absorption peak near 268nm for Ti_0.91_O_2_/CdS films, which is ascribed to the contribution from Ti_0.91_O_2_. The type II band alignment between CdS and Ti_0.91_O_2_ is favorable for transfer of photogenerated electrons from CdS to Ti_0.91_O_2_.


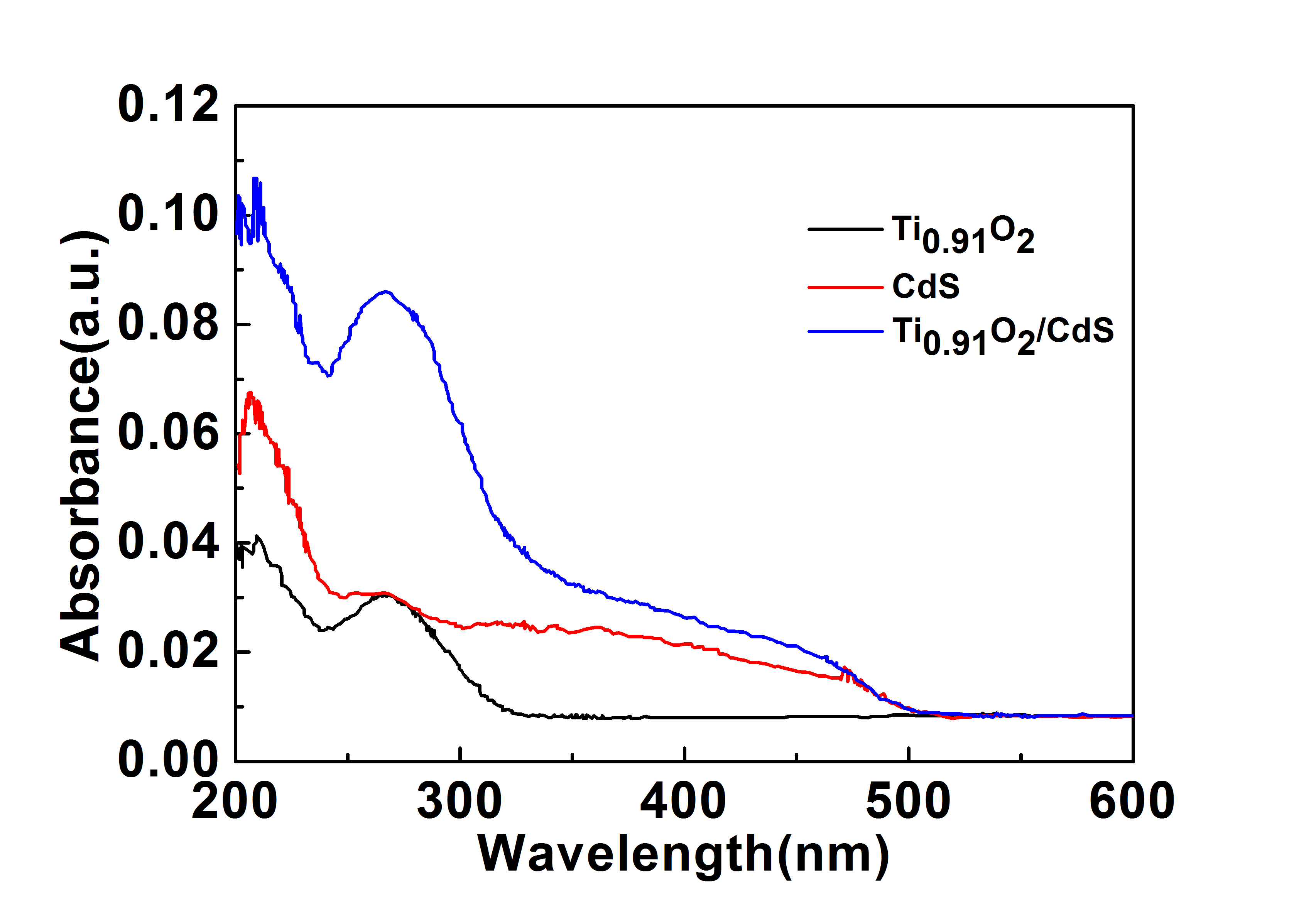


Figure S1. UV-vis absorbance spectra of Ti_0.91_O_2_, CdS and Ti_0.91_O_2_/CdS.(supported by Tu WG)

Compared with Raman spectra of Ti_0.91_O_2_ in Figure S2, the optical mode at around 300 cm^-1^, together with its overtones at 600 cm^-1^ and 900cm^-1^ for CdS is observed in the spectra of all Ti_0.91_O_2_/CdS, showing a combination of two semiconducting characteristic bands.

**
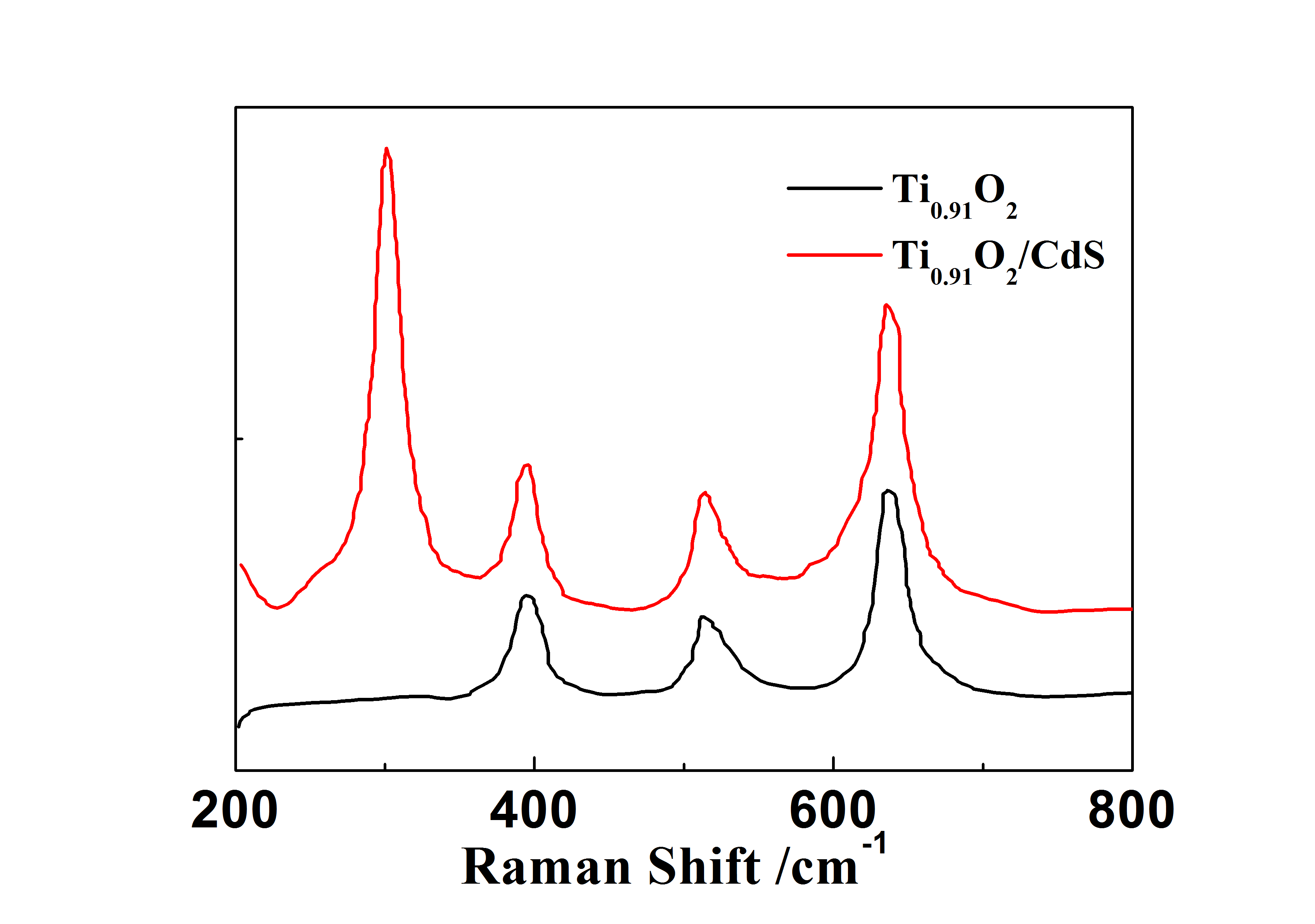
**

Figure S2. Raman spectra of pure Ti_0.91_O_2_, Ti_0.91_O_2_/CdS.


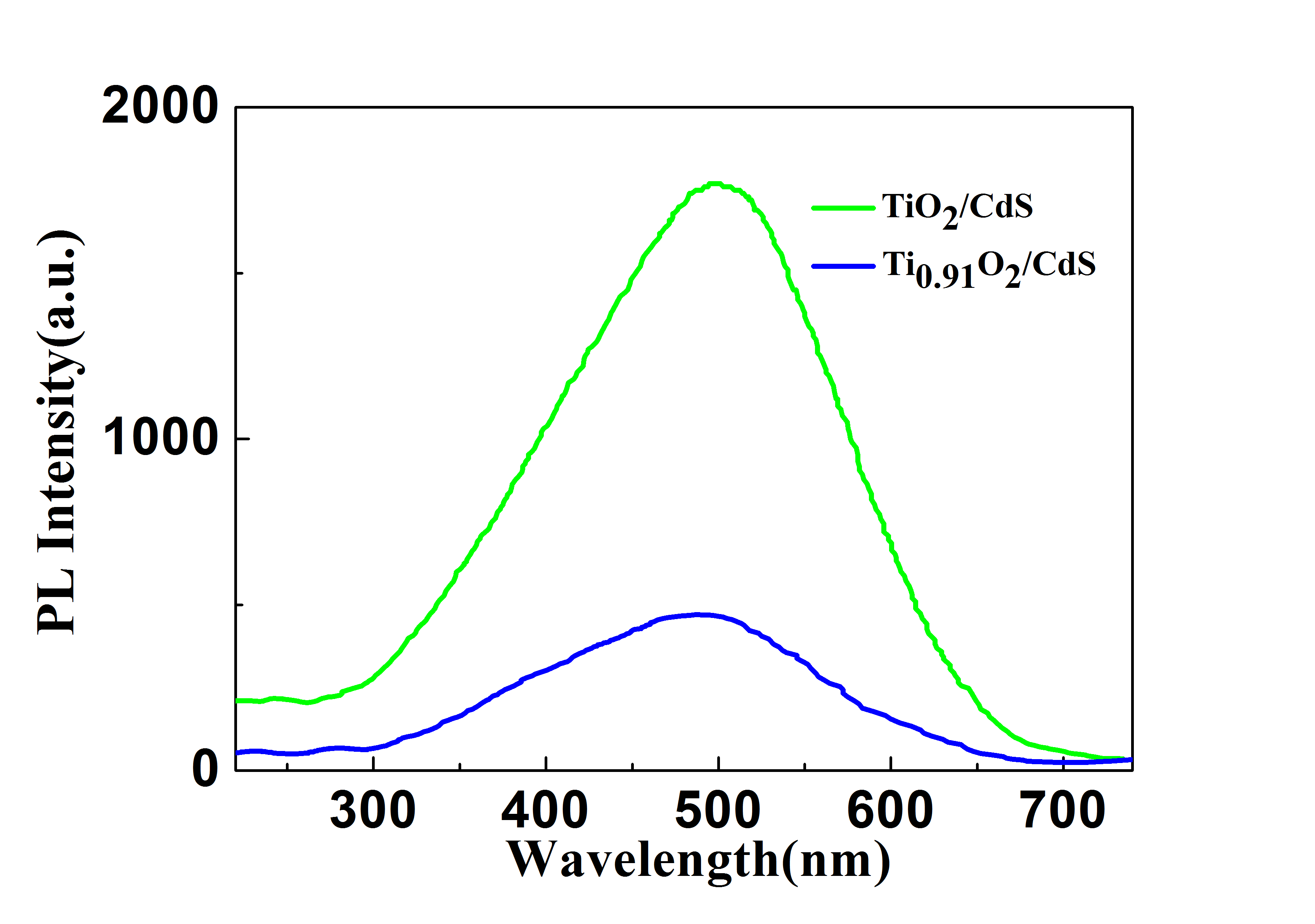

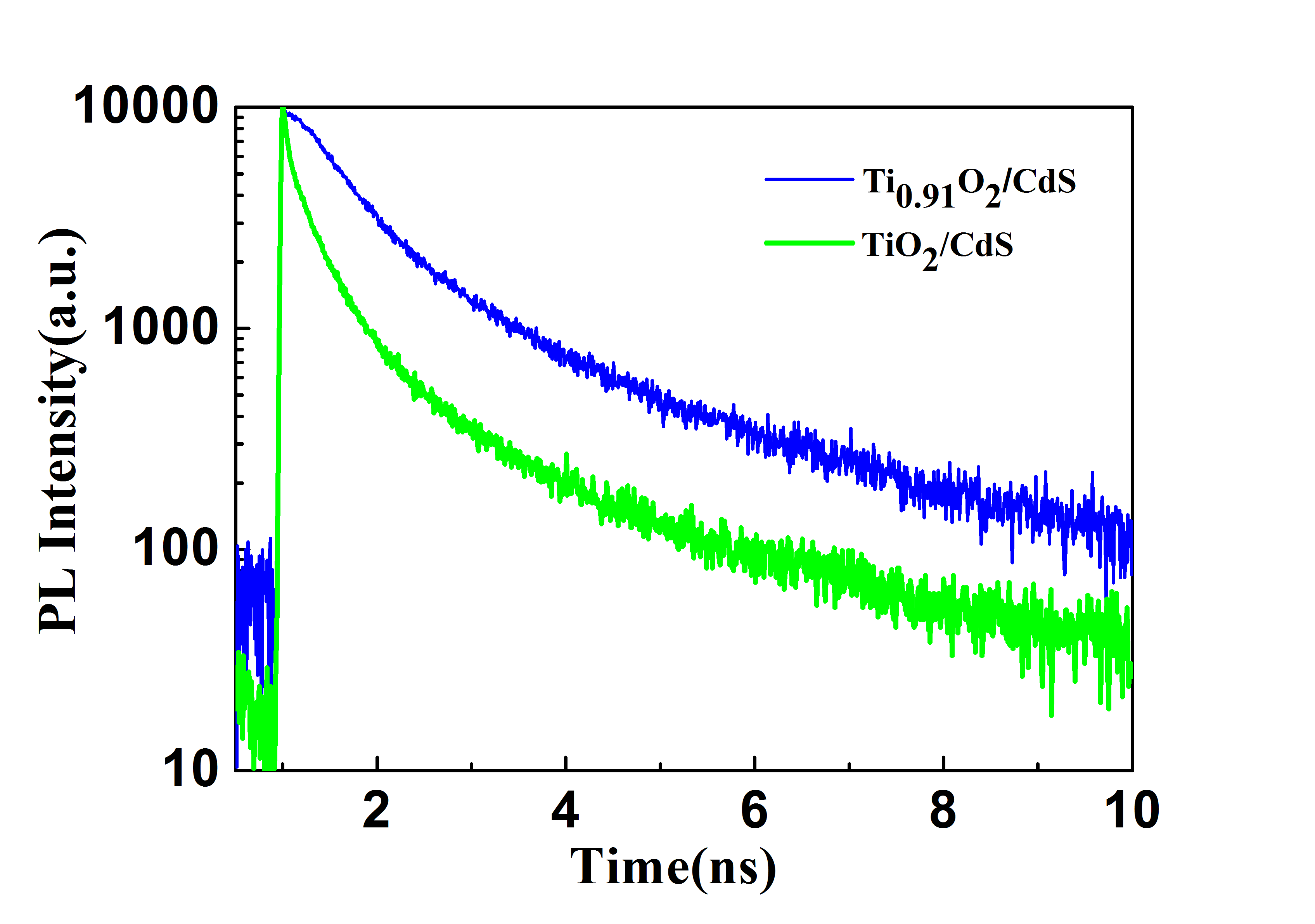


Figure S3(a). PL spectra of the Ti_0.91_O_2_/CdS and Ti_0.91_O_2_/CdS; (b) PL decay curves of the TiO_2_/CdS and Ti_0.91_O_2_/CdS.

To better compare charge transfer and electronic interaction between Ti_0.91_O_2_/ CdS and TiO_2_/CdS, PL spectra and transient time-resolved PL decay measurements were carried out on the samples Ti_0.91_O_2_/CdS and TiO_2_/CdS excited at 266 nm laser wavelenght. Under the excitation of 266 nm, TiO_2_/CdS shows an emission peak around 500nm as shown in Figure S3(a). Compared with TiO_2_/CdS spheres, the emission peak of Ti_0.91_O_2_/CdS spheres shows the same emission peak, which is nearly consist with the bandgap value between valence band of CdS NPs and the conduction band of Ti_0.91_O_2_ nanosheets. However, transient PL decay traces of TiO_2_/CdS hollow spheres show short PL lifetime of 2.4ns. In contrast, the ultrathin Ti_0.91_O_2_/CdS hollow spheres displays a longer PL decay lifetime of 3.6ns as shown in Figure S3(b). The prolonged decay lifetime observed in Ti_0.91_O_2_/CdS hollow spheres reveals that decay dynamics for Ti_0.91_O_2_/CdS hollow spheres are fundamentally different from traditonal TiO_2_/CdS system.


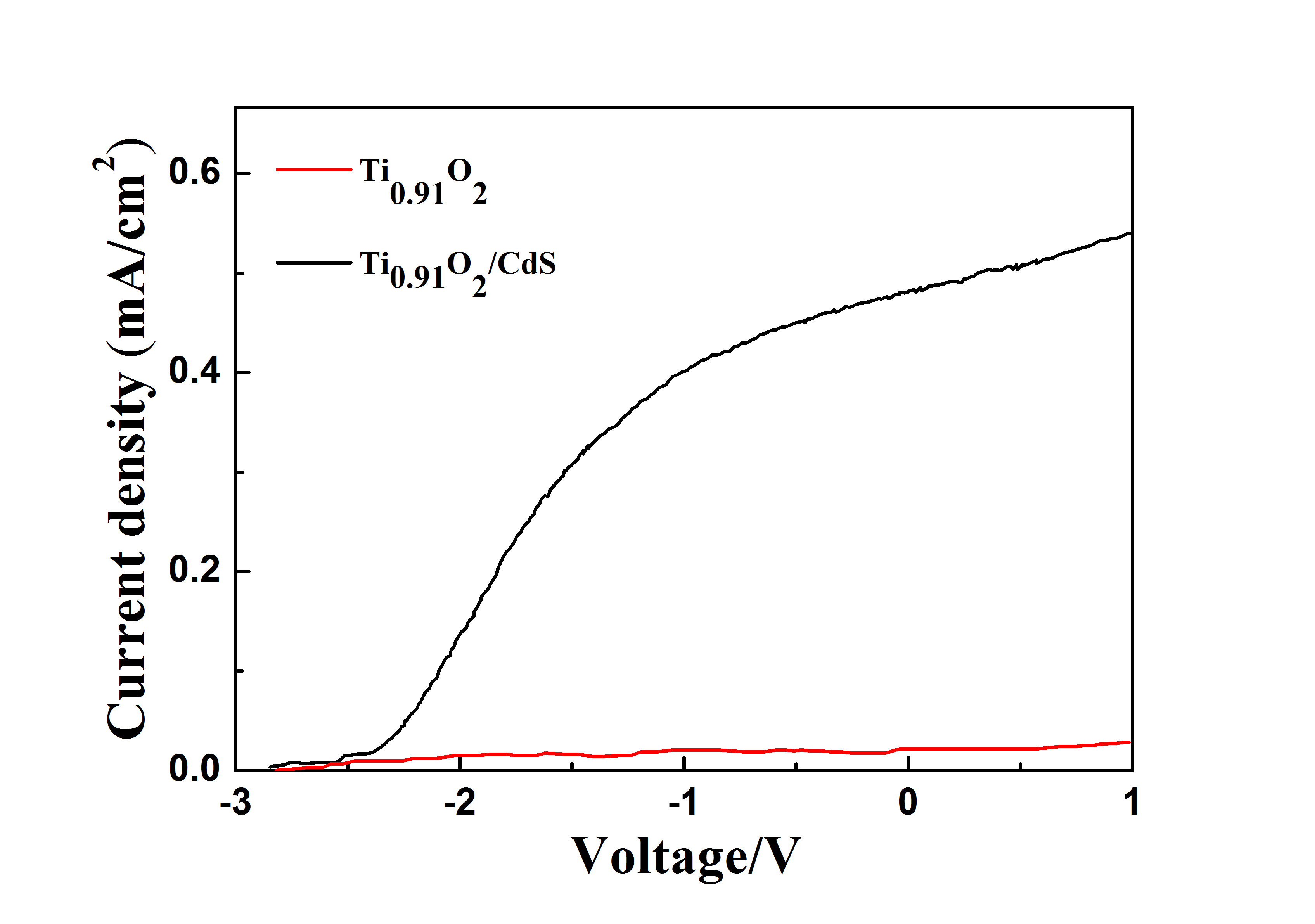


Figure S4. J−V curves of the Ti_0.91_O_2_, and Ti_0.91_O_2_/CdS

To characterize the ability of the samples, linear J−V curves were recorded as shown in Figure S4. The great enhancement of the photocurrent after CdS sensitization shows the advantage of the Ti_0.91_O_2_/CdS compared to the Ti_0.91_O_2_ with light illumination. Therefore, a higher loading of the photosensitizer will lead to a higher photocurrent density.
